# Sternalis Syndrome Misidentified by Multiple Specialties Responding to Botox Treatment: A Case Report and Literature Review

**DOI:** 10.7759/cureus.42236

**Published:** 2023-07-21

**Authors:** Kateryna Georgiyeva, Harendra Kumar, Vania E Fernandez

**Affiliations:** 1 Internal Medicine, Memorial Healthcare System, Pembroke Pines, USA; 2 Medicine and Surgery, Dow University of Health Sciences, Karachi, PAK; 3 Pain Management, Broward Spine Institute, Hollywood, USA

**Keywords:** unusal causes of persistent chest pain, atypical chest pain, exertion chest pain, non-cardiac chest pain, botox injections, sternalis syndrome

## Abstract

When a clinician, especially one who is specialized in primary care is faced with presenting complaints of deep, sharp, anterior chest pain, the most common differential diagnoses include cardiac and gastrointestinal pain. Musculoskeletal pain is thought of less frequently as a possible root cause. In this case report, we describe the clinical journey of a female athlete who presented with complaints of burning anterior chest pain. Her sternalis syndrome pain was first misdiagnosed as pain of cardiac origin, resulting in pacemaker placement into the patient’s chest. The pain continued, and the same musculoskeletal pain was then presumed to be of gastrointestinal origin due to a previous history of gastroesophageal reflux disease (GERD). As a result of this misidentification, the patient underwent an unnecessary esophageal surgical procedure. Here, we identify the origins of sternalis syndrome pain, what other conditions the pain may be confused with, and how clinicians should not be quick to exclude musculoskeletal pain from a differential diagnosis of acute chest pain. We discuss effective treatments for sternalis syndrome and shed light on this less common cause of anterior chest pain to promote more accurate diagnosis and avoidance of unnecessary surgical interventions.

## Introduction

Sternalis syndrome is a rare and often misdiagnosed condition characterized by severe chest pain localized to the sternum, with the prevalence of the sternalis muscle being detected in 7.8% of the general population [1]. It is caused by abnormal muscle fibers known as sternalis muscles, which are variably present in the anterior chest wall [1]. These accessory muscles, originating from the pectoralis major or rectus abdominis muscles, can become hyperactive or undergo spasms, leading to intense pain in the sternum region [1-2]. The clinical presentation of sternalis syndrome closely mimics cardiac conditions, musculoskeletal disorders, and other causes of chest pain, making an accurate diagnosis challenging [2].

In this case report and literature review, we present the remarkable journey of a 51-year-old female marathon runner who suffered from excruciating deep sternal chest pain for several years. Despite seeking medical help and undergoing evaluations from multiple specialties, her symptoms remained unexplained and unrelieved. The diagnostic process involved extensive cardiac evaluations, nerve blocks, and joint injections, all of which failed to provide a definitive diagnosis or alleviate her pain. After a comprehensive evaluation and diligent assessment, sternalis syndrome was identified as the underlying cause of the patient's persistent chest pain. This diagnosis was made possible by recognizing the unique clinical features and distinguishing them from other potential causes. Subsequently, a treatment plan involving Botox injections targeting the hyperactive sternalis muscles was initiated, leading to significant and sustained pain relief.

Treatment for sternalis syndrome typically involves a multimodal approach aimed at relieving pain and improving functional outcomes [2-3]. Conservative measures such as nonsteroidal anti-inflammatory drugs (NSAIDs), physical therapy, and targeted exercises may be employed to manage symptoms [3]. Local interventions, including injections of local anesthetics and corticosteroids into the sternomanubrial joint or surrounding structures, can provide temporary pain relief [2-3]. In cases of refractory pain, Botox injections have shown promise by temporarily paralyzing the hyperactive sternalis muscles. However, the optimal treatment approach for sternalis syndrome is yet to be established, and individualized plans tailored to the patient's specific symptoms and response to interventions are crucial. Collaboration among healthcare professionals from various disciplines is essential to ensure comprehensive care and optimize treatment outcomes [3].

This case report not only emphasizes the importance of considering sternalis syndrome as a potential cause of chest pain but also highlights the effectiveness of Botox injections in managing the condition. Furthermore, it emphasizes the significance of multidisciplinary collaboration and a patient-centered approach to navigate complex cases like sternalis syndrome. Ultimately, this report contributes to the growing body of literature on sternalis syndrome and sheds light on the potential for successful outcomes when the condition is accurately identified and treated with Botox injection.

## Case presentation

A 51-year-old female marathon runner presented in May 2019 with excruciating deep sternal chest pain that she had been experiencing since 2014. On a scale of 0-10, commonly used by practitioners in the United States to assess pain, the pain was rated 10/10, and was described by the patient as excruciating. The pain was specifically triggered by exercise, especially running. At rest, the patient did not have any significant symptoms. NSAIDs provided no relief for her pain. In 2017, the patient had undergone a cardiac stress test, and additional testing which revealed an atrioventricular (AV) conduction issue. Despite the belief that this issue was not the cause of her pain, a pacemaker was implanted, rendering her pacemaker dependent on both the atria and ventricles. The patient also had a hiatal hernia repair in 2017, accompanied by a sphincter augmentation procedure using an artificial gastrointestinal sphincter. Unfortunately, even after these interventions, the patient continued to experience the same severe chest pain. In May 2019, bilateral sternochondral joint injections were performed with an initial diagnosis of presumed sternochondral chondrodynia.

However, the injections provided minimal pain relief. In June 2019, a midline manubriosternal joint injection was administered, also resulting in minimal relief. Further attempts to alleviate the patient's pain included bilateral sternochondral joint injections at the T5, T6, and T7 levels in July 2019, using lidocaine and steroid solution. Despite these interventions, the patient reported partial relief from ketorolac for breakthrough pain and found some relief from chiropractic thoracic spine adjustments and a diagnostic block of the costosternal junction. Various nerve blocks were performed, including a right and left intercostal nerve block at the T7 level, but none provided lasting relief. The most effective treatment at that point was a sternal block, which provided significant pain relief. As a potential long-term solution, the patient underwent chemoneurolysis of nerves in the affected sternal region of the midline sternomanubrial joint. In October 2019, alcohol chemoneurolysis was performed on the intercostal nerve branches of the sternomanubrial joint. This intervention resulted in a 70% improvement in pain, but the patient still experienced residual intercostal neuralgia symptoms in the upper thoracic levels. 

**Figure 1:**
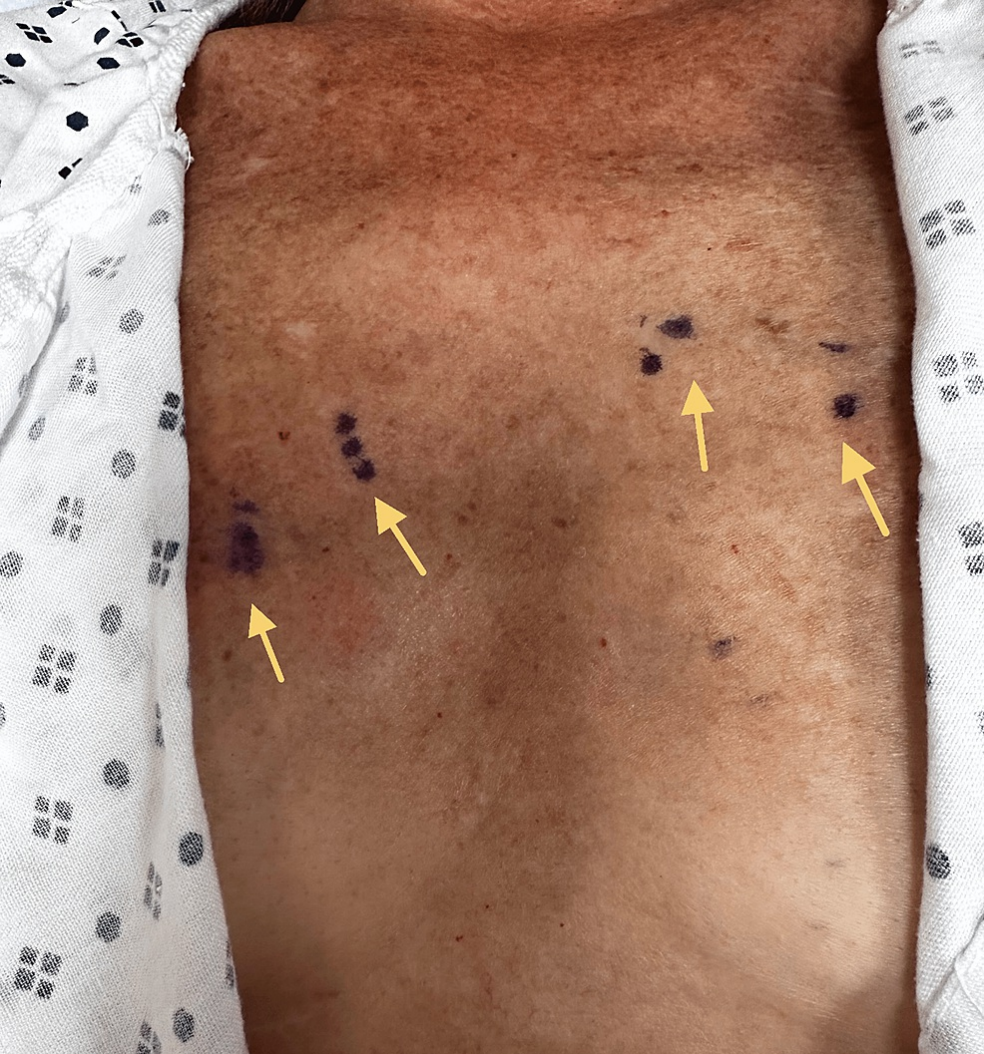
Points of Botox injection on both sides of the patient's sternum are shown (yellow arrows).

**Figure 2:**
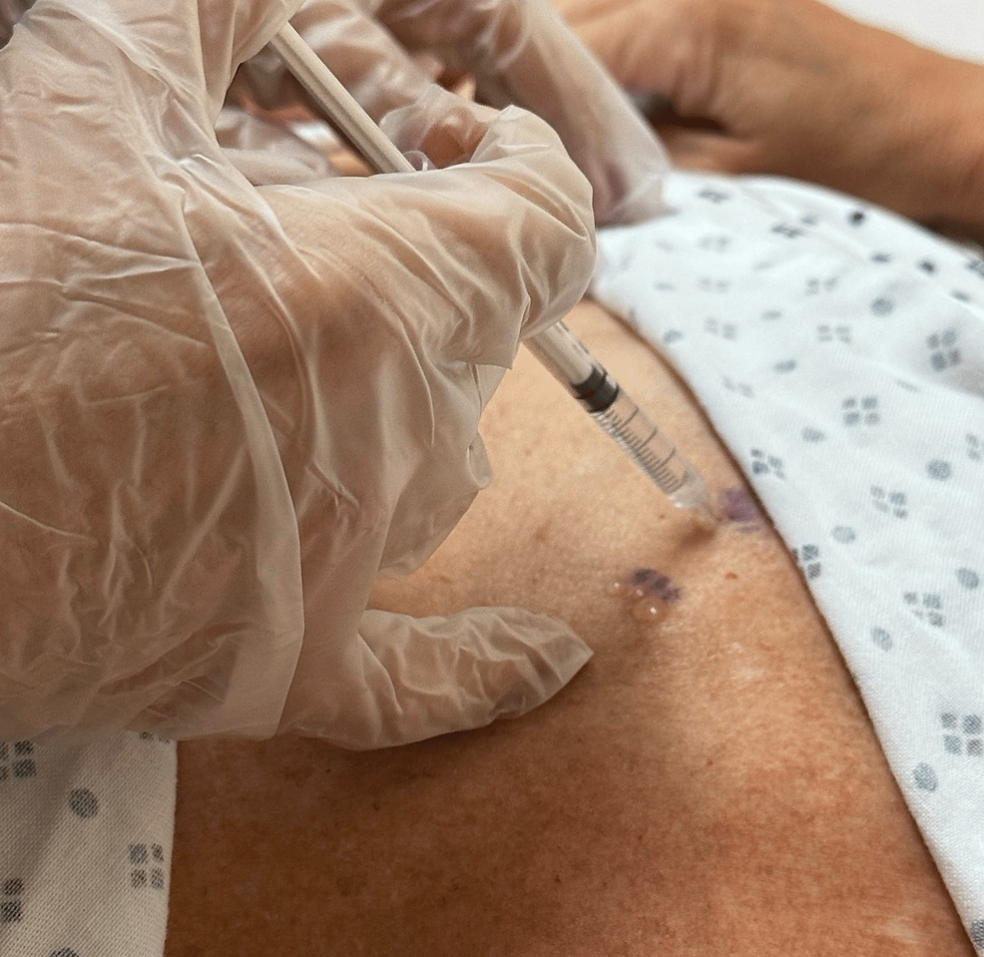
Botox injection into a painful point in the sternalis muscle by a pain management physician is depicted.

A diagnostic nerve block was performed at the T2, T3, and T4 levels, targeting the left T4 level, which radiated pain toward the anterior chest and myocardium. However, this block did not provide lasting pain relief. In November 2019, the patient's Medtronic pacemaker was temporarily deactivated to facilitate an MRI, but this intervention did not yield any helpful information. In January 2020, the working diagnosis was sternalis syndrome, characterized by spasms of the sternalis muscle. Botox injections were recommended as a treatment, and proved to be effective, confirming this diagnosis. The patient received midline sternomanubrial joint and bilateral sternalis muscle injections using Depo Medrol and Bupivacaine in January 2020. Subsequent Botox treatments were administered to the sternalis muscles of the anterior chest. The patient reported a good effect after the second Botox treatment, with a decrease in pain intensity and duration, particularly during activity. The patient also received Botox treatment for chronic refractory migraines and cervical dystonia. By August 2020, the patient reported a decrease in the frequency, intensity, and duration of pain in the previous three months. A bilateral intercostal nerve block was performed on the left anterior chest to block the anterior cutaneous branches as a treatment for musculoskeletal chest pain. Additionally, the patient underwent a fourth Botox treatment, which further contributed to pain reduction. Figure 1 depicts points on bilateral sides of the patient's sternum where chest pain was felt. Using a medical marking pen, points of injection were identified, and Botox was injected directly into the most painful points on the chest wall, as shown in Figure 2.

By November 2020, the patient had failed medical management with several classes of prescription drugs, leading to another Botox injection. The patient continued to experience a decrease in pain. In February 2021, there was a further reduction in pain, and the patient was able to maintain improved results. She was able to resume regular physical activity, even successfully completing a marathon in April 2021. Pain relief has been maintained since with a Botox regimen of 100 units every six weeks. As of the latest follow-up, the patient remains pain-free.

## Discussion

Sternalis syndrome, although a relatively rare condition, presents a diagnostic challenge due to its resemblance to other musculoskeletal and cardiac disorders [1-2]. This case report highlights the misidentification of sternalis syndrome by multiple specialties and the favorable response to Botox treatment. In this discussion, we will explore the clinical presentation, diagnostic considerations, treatment modalities, and role of Botox in managing sternalis syndrome, along with a review of relevant literature.

Sternalis syndrome is characterized clinically by severe deep sternal chest discomfort that is generally exacerbated by exercise or increased heart contractions [2]. This distinct pattern of discomfort, which occurs only during physical activity, separates it from other forms of chest pain, such as cardiac ischemia or musculoskeletal injuries [2]. The case presented here illustrates the patient's history of suffering significant pain when running a long-distance marathon while being asymptomatic at rest. When assessing patients with exercise-induced chest pain, these specific symptoms should highlight the possibility of sternalis syndrome.

Despite its distinct clinical presentation, sternalis syndrome is often misdiagnosed or overlooked by healthcare providers. In this case, the patient underwent many diagnostic tests and treatments, including heart stress testing, pacemaker installation, hiatal hernia surgery, and repeated joint injections, without achieving substantial pain relief. This emphasizes the need to include sternalis syndrome in the differential diagnosis of exercise-induced chest pain, particularly when standard diagnostic methods fail to provide relief.

A thorough clinical assessment, a thorough physical examination, and the exclusion of other possible causes of chest pain are required for an accurate diagnosis of sternalis syndrome [3]. To rule out cardiac or musculoskeletal disorders, imaging methods such as CT scans and MRI may be conducted [4]. The gold standard for diagnosis, however, remains the identification of sensitive areas and replication of pain with sternal muscle palpation [4-5]. Clinical observations, a physical examination, and the patient's response to Botox treatment were used to corroborate the definitive diagnosis in this case.

Sternalis syndrome treatment comprises a multimodal approach tailored to the symptoms and preferences of the particular patient. Conservative treatments, such as NSAIDs, physical therapy, and targeted exercises, may provide relief in mild cases or as additional therapy [5-6]. Interventional therapy, on the other hand, may be required in circumstances where conservative techniques have failed [7]. In this case, the patient had many joint injections, nerve blocks, and a diagnostic and therapeutic midline block of the sternomanubrial joint, all of which provided instant relief but did not result in long-term pain management.

The positive response to Botox treatment in sternalis syndrome is a notable feature of this case. Botox, a neurotoxin derived from Clostridium botulinum, causes muscle relaxation by temporarily blocking the release of acetylcholine at the neuromuscular junction [8]. Botox has gained popularity in the treatment of sternalis syndrome due to its ability to minimize muscular spasms and pain [8-9]. In this case, Botox injections into the sternomanubrial joint and sternalis muscles reduced pain intensity and frequency significantly, allowing the patient to resume regular physical activity, including marathon running.

Botox's method of action in sternalis syndrome is not well understood [10]. However, it is proposed that the hyperactive sternalis muscle, which contributes to patient discomfort, is temporarily paralyzed, leading to muscular relaxation and pain relief [10-11]. Botox injections are a less intrusive and reversible therapeutic option for those with refractory sternalis syndrome, particularly when other treatments have failed [11].

While Botox has shown promising results in the treatment of sternalis syndrome, it is important to note that its use should be approached with caution. Potential side effects, such as muscle weakness, injection site reactions, and allergic reactions, should be discussed with the patient, and the risks and benefits should be carefully balanced [11-12]. Furthermore, the optimal dosage, injection technique, and frequency of Botox injections in sternalis syndrome have not yet been established. More research and larger clinical trials are required to provide evidence-based recommendations for the use of Botox in this context [12].

Sternalis syndrome is a rare but important cause of exercise-induced chest pain that might be misconstrued by various specialties. Issues such as cardiac angina, fibromyalgia, GERD, and costochondritis should be included in the differential diagnosis list, as they are more common causes of exercise-induced chest pain. This case report emphasizes the need to detect sternalis syndrome clinical signs and include them in the differential diagnosis of patients presenting with exercise-induced chest pain. Conservative treatments and interventional techniques may provide temporary relief; however, Botox injections have shown promising success in managing refractory sternalis syndrome.

## Conclusions

Sternalis syndrome, although often misidentified or overlooked, is an important consideration in the evaluation of exercise-induced chest pain. This case report highlights the diagnostic challenges faced by multiple specialties and the favorable response to Botox treatment in managing sternalis syndrome. The clinical presentation, diagnostic considerations, and treatment modalities discussed in this report shed light on the complexity of this condition and the need for a multidisciplinary approach. Moreover, sternalis syndrome poses a diagnostic challenge due to its resemblance to other musculoskeletal and cardiac disorders. This case report highlights the importance of recognizing the clinical features of sternalis syndrome and considering it in the differential diagnosis of exercise-induced chest pain. Collaboration among healthcare professionals from various specialties is crucial to accurately diagnosing and managing this condition. While conservative measures and interventional treatments may provide temporary relief, Botox injections have shown promise in achieving significant and sustained pain reduction. Further research is warranted to enhance our understanding of sternalis syndrome and optimize treatment approaches for better patient outcomes.
